# Effects of different adding methods of fermentation medium on the quality of cigar

**DOI:** 10.3389/fbioe.2024.1440961

**Published:** 2024-08-12

**Authors:** Cai Wen, Yang Shuanghong, Hu Wanrong, Chen Ran, Chai Zhishun, Huo Danqun, Hou Changjun, Li Dongliang, Zhang Qianying

**Affiliations:** ^1^ Key Laboratory for Biorheological Science and Technology of Ministry of Education, Bioengineering College of Chongging University, Chonging, China; ^2^ Cigar Fermentation Technology Key Laboratory of China Tobacco, China Tobacco Sichuan Industrial Co., Ltd., China Tobacco Technology Innovation Center for Cigar, Chengdu, China; ^3^ Technology Center, Guangdong China Tobacco Industry Co., Ltd., Guangzhou, China

**Keywords:** fermentation medium, cigar, microorganisms of tobacco, plant extracts, bacterial agents, chemical composition

## Abstract

**Background:** To investigate the effects of different media and media addition methods on the fermentation of tobacco.

**Methods:** A plant extract and a bacterial agent are used for fermenting cigar tobacco leaves in a different order of addition. The chemical composition, cellulose, and pectin content, as well as changes in the microbial community, were measured.

**Results:** The addition method of the fermentation medium affects the quality of fermented tobacco. The optimal medium formula and addition method involve first adding plant extracts and then microbial agents. The medium formula and addition method can significantly reduce cellulose in tobacco, with a reduction rate of 46%, and significantly increase the content of β-carotene, thereby enhancing the aroma of cigarettes. There is an increase in aroma components, such as alcohols, alkanes, and olefins, in tobacco. By reducing the proportion of Aspergillus, it can alter the microbial community structure of tobacco.

**Conclusion:** Adding plant extracts before introducing microbial agents can significantly improve the quality and alter the microbial community structure of Dexue No.1 tobacco.

## 1 Introduction

The cigar is a type of tobacco product with a strong aroma and unique flavor. Fermentation plays a crucial role in determining the quality of cigar tobacco leaves. Appropriate fermentation methods and conditions can significantly enhance the quality of fermented cigar tobacco leaves (Lei, 2022; Zhang et al., 2023b). The traditional water fermentation method effectively addresses the quality deficiencies of domestic cigar tobacco, such as lack of maturity, poor aroma, and insufficient flavor. Introducing additional substances into the cigar tobacco leaves for medium fermentation can significantly enhance the smoking characteristics of the leaves and modify their chemical composition, flavor compounds, and microbial community structure. Therefore, it is common practice for factories to employ medium-assisted further fermentation treatment for cigar tobacco following the initial fermentation process.

The commonly used cigar tobacco fermentation medium mainly includes plant extracts, microbial agents, and enzyme preparations (Ling and Zhang, 2016; Zheng et al., 2022; Yahao et al., 2023; Yong et al., 2023; Zhang et al., 2023b). After fermentation with the medium, the sensory quality of cigar tobacco is significantly improved. However, different mediums can improve cigar quality from different angles. For example, cocoa (Hu et al., 2023a), rice wine (Henduo et al., 2023), and glutinous rice water (Zhang et al., 2023b)plant extracts mainly enhance the sensory quality of cigar tobacco by boosting the aroma quality and aroma intensity. On the other hand, adding *tremella aurantialba* (Zhang et al., 2023a), *pseudomyces* (Jia et al., 2023), *protease* (Kou et al., 2011), and *cellulase* (Ju et al., 2023) microbial agents and enzyme preparations to ferment cigar tobacco mainly improves the sensory quality by reducing the off-flavors and stimulants.

Currently, the fermentation media for cigars mainly consist of microbial agents, enzyme preparations, and plant extracts. Hu et al. investigated the influences of diverse media, predominantly various types of plant extracts, on the chemical composition and sensory quality of tobacco leaves (Hu et al., 2023a)^.^. Zhang et al. employed golden ear to ferment cigar leaves with eggplant core and discovered that the sensory quality of the cigar leaves was enhanced (Zhang et al., 2023a). Jia et al. utilized the microbial agent prepared by *candida* yeast to ferment cigar tobacco leaves to improve the quality of the tobacco leaves (Jia et al., 2023). Kou and Ju et al. used exogenous enzyme preparations as media to participate in the fermentation process of tobacco leaves. Previous studies mostly concentrated on the research and development of a single new medium and its mechanism of action (Kou et al., 2011; Ju et al., 2023). Few studies were carried out on the compounding of different media and its addition process.

In this study, the effects of adding different orders on the quality of cigar tobacco leaves were investigated. The changes in total sugar, reducing sugar, cellulose, pectin, plastid pigment, and microorganisms in cigar tobacco leaves were measured. Simultaneously, sensory evaluation was conducted to offer new insights and technical support for enhancing the quality of fermented cigar tobacco. Previous studies have mostly focused on the fermentation of a single medium. This study is the first to utilize mixed fermentation of two media to optimize the addition method. It also explores the changes in microbial community and key active enzyme gene content during the process, with the aim of providing technical support for the widespread adoption and utilization of composite media.

## 2 Materials and methods

### 2.1 Materials

Decue No. One cigar leaf, natural plant extract and bactericide for fermentation, provided by Sichuan China Tobacco Industry Co., Ltd. Changcheng Cigar Factory.

### 2.2 Methods

#### 2.2.1 Fermention

After Dexue No. 1 tobacco leaves are moistened and mixed, natural plant extracts and bactericides are evenly sprayed on the surface of tobacco leaves according to the treatment method in Table 1, and the leaves are put into fermentation room for fermentation after there is no clear water on the surface of the tobacco leaves. Tobacco fermentation condition is 35°C, humidity 75% The pH environment of the tobacco leaf itself is slightly acidic and neutral. The pH value of the plant extract is 3.75 ± 0.03, and the pH value of the bactericide is 4.45 ± 0.02. Due to the small amount of medium added, it will not cause significant fluctuations in the pH of the tobacco leaves. The pH values of untreated raw tobacco leaf and fermented leaf are shown in the table below.

**TABLE 1:** The adding method of fermentation medium.

Number	Processing method
JM1	Add only microbial agents
JM2	Add only plant extracts
JM3	Bacteria and plant extracts are added together
JM4	Add bacteria first, then add plant extracts
JM5	Add plant extracts first, then add bacteria
DX1	Untreated

#### 2.2.2 Sensory evaluation

The tobacco leaf samples were ventilated and dried, and the moisture content was (15 ± 0.5) % after moisture regain. The tobacco leaves after moisture regain were hand-rolled into single-material tobacco samples. The smoking group composed of sensory evaluation experts identified and scored the quality characteristics of cigar leaves, and scored them according to 0–9 points. The quality characteristics of cigar leaves include four aspects, including aroma characteristics such as aroma quantity, richness and maturity, smoke characteristics such as irritation, softness and fineness, aftertaste characteristics such as sweetness, cleanliness and aftertaste, and combustion characteristics such as flammability, grayness and condensation grayness.

#### 2.2.3 Chemical composition analysis

Continuous flow analysis (Zhu et al., 2023) was used to determine total sugar and reducing sugar in cigar tobacco samples (YC/T 159–2002). The contents of cellulose and pectin were determined by kit. The plastid pigment of cigar tobacco was determined by high performance liquid chromatography (YC/T 382–2010). The volatile components of cigar tobacco were determined by GC-MS (Hu et al., 2023b).

#### 2.2.4 Microbiological analysis

Weigh 8g tobacco leaves and grind it into powder in a mortar. Add the tobacco leaf powder into a conical bottle containing sterile normal saline and place it in a shaker for shock. After the shock, filter it with sterile gauze, collect filtrate and centrifuge to obtain bacterial mud. Microbial metagenomic extraction, quality control, library construction and computer support is provided by Megi Biology.

#### 2.2.5 Data processing and analysis

Single factor ANOVA test was performed on the data using SPSS22, and the letter labeling of significant differences between groups was mainly based on Waller-Duncan results in homogeneous subsets. Draw histogram with OriginPro 2021; Use graphpad prism9.0.0 to draw heat maps; Plot the bubble using ggplot2.

## 3 Result

### 3.1 Sensory evaluation

**FIGURE 1:**
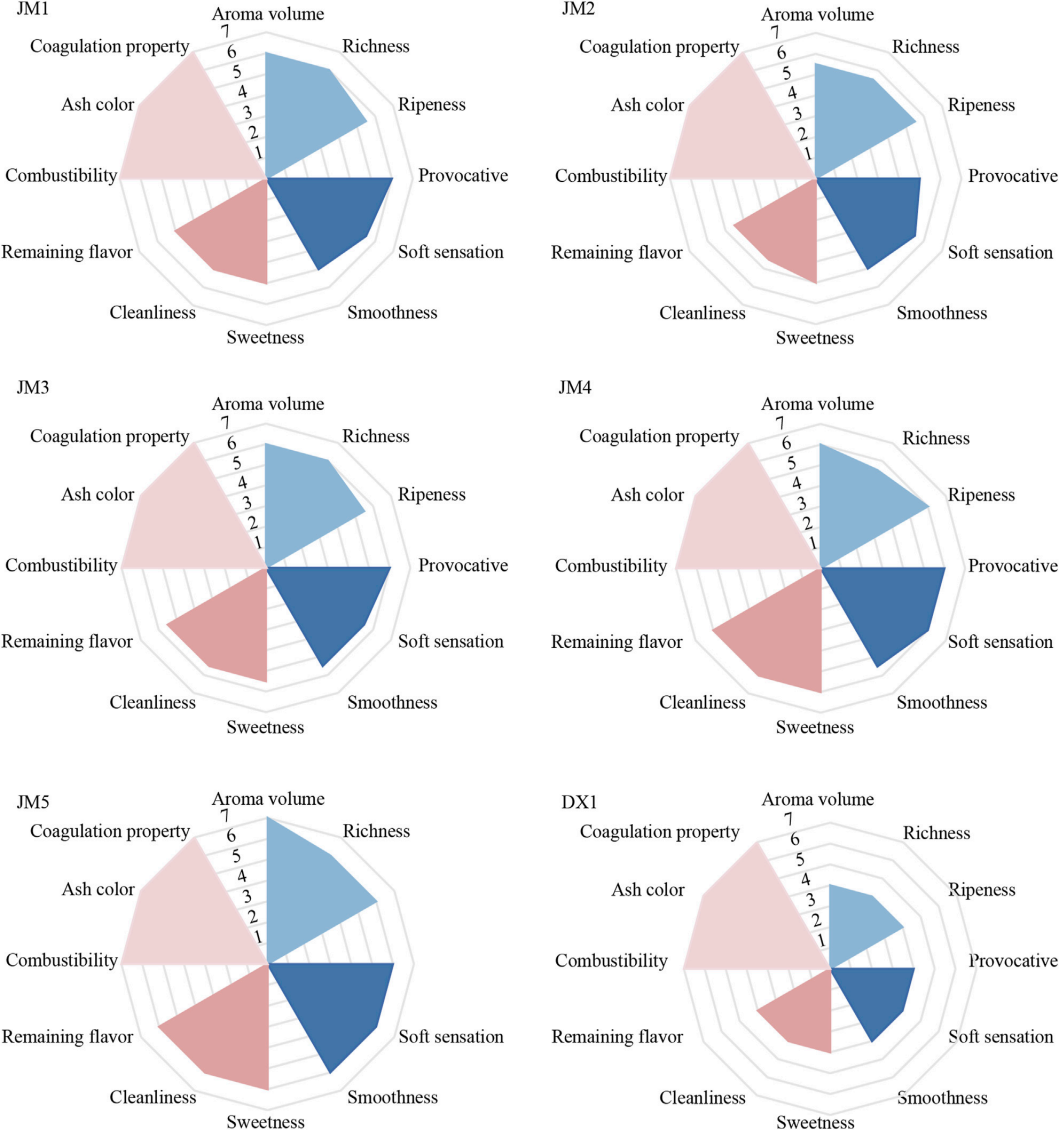
Sensory evaluation radar chart. Note: The evaluation results were based on untreated tobacco DX1 as a control.

After the fermentation of tobacco leaves, the leaves were rolled into individual cigar. These cigar were then assessed by experts based on four dimensions. The relevant sensory evaluation results are shown in Figure 1. Compared with untreated tobacco leaves (DX1), the aroma indexes of tobacco leaves improved after fermentation with added media. The aroma quantity of JM1 was greater than that of JM2, the irritation of JM2 was lower, and JM3 was superior to both JM1 and JM2. The sensory evaluation scores of JM3, JM4, and JM5 increased sequentially. The sensory evaluation results of JM5 were outstanding, primarily characterized by a rich aroma, smooth and clear smoke, and sweetness.

### 3.2 Conventional chemical composition

**FIGURE 2:**
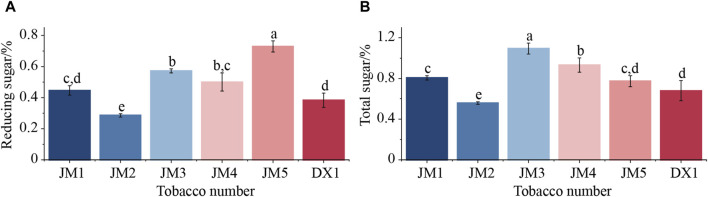
Mass fractions of reducing sugars and total sugars in tobacco. Note: Lowercase letters indicate significant differences between groups, *p* < 0.05. Note: **(A)** Reducing sugars of cigar tobacco leaves, **(B)** Total sugars of cigar tobacco leaves.

The total sugar and reducing sugar content of cigar tobacco leaves were determined using continuous flow analysis (Figure 2). The results showed that the effects of different mediums and addition methods on reducing sugar and total sugar in tobacco leaves varied. It can be seen from Figure 2 that when only one medium was added, the levels of reducing sugar and total sugar in JM2 fermented with plant extracts were significantly lower than those in the untreated sample. This could be attributed to the plant extracts promoting the growth of microorganisms, leading to an increase in the metabolic utilization of reducing sugar and a decrease in reducing sugar content in tobacco leaves. The total sugar in JM1 fermented by adding microbial agents increased significantly, while the reducing sugar did not change significantly. This suggests that the total sugar content was elevated due to the introduction of additional non-reducing sugars through the addition of microbial agents. When the two mediums were added together, reducing sugar increased significantly. This suggests that combining the two mediums was beneficial for microbial metabolism, leading to an increased production of reducing sugar. At the same time, there were differences between JM3, JM4, and JM5, indicating that the addition of the two mediums had an effect on the formation of reducing sugar.

### 3.3 Changes of plastid pigment content in cigar tobacco leaves

**FIGURE 3:**
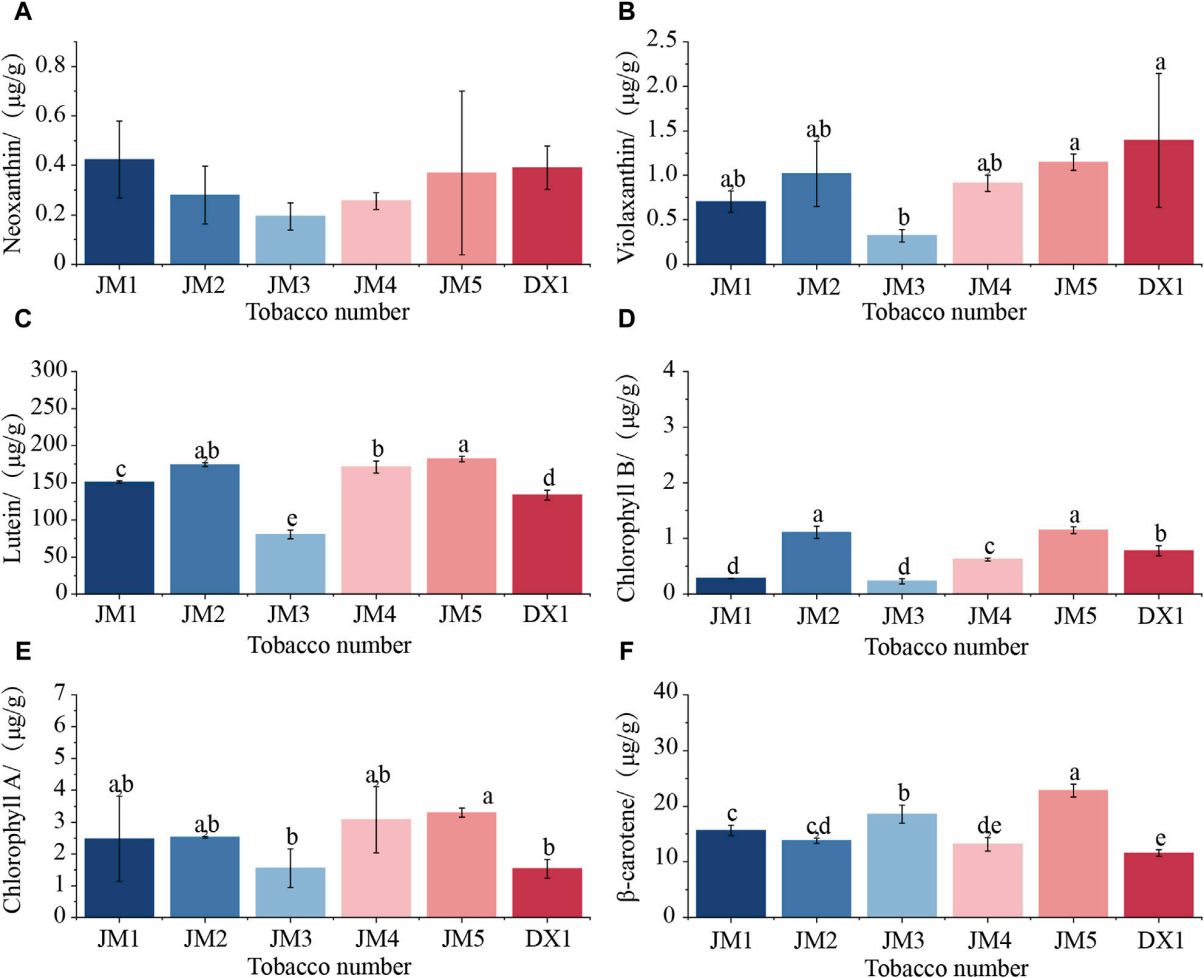
Changes in plastid pigment content in cigar leaf samples. **(A)** Neoxtanthin of cigar tobacco leaves; **(B)** Violaxanthin of cigar tobacco leaves; **(C)** Lutein of cigar tobacco leaves; **(D)** Chorophyll B of cigar tobacco leaves; **(E)** Chorophyll A of cigar tobacco leaves; **(F)** β-carotene of cigar tobacco leaves.

It can be seen from Figure 3 that the content of plastid pigment in tobacco leaves changed to varying degrees after adding medium for fermentation, and the change in neoxanthin was not significant. Compared with DX1, the levels of lutein and β-carotene in JM1 significantly increased, while the amount of chlorophyll B decreased significantly. The levels of lutein, chlorophyll B, and β-carotene in JM2 showed a significant increase. When the two mediums were added separately, both of them had positive effects on lutein and β-carotene content, and negative effects on chlorophyll B content. When the two medium were combined, the levels of violaxanthin, lutein, and chlorophyll B in JM3 decreased significantly, whereas the amount of β-carotene increased. The lutein content in JM4 increased significantly, while the levels of other plastid pigments did not change significantly. The levels of lutein, chlorophyll A and B, and β-carotene in JM5 increased. The addition method of the two mediums directly affects the synthesis of plastid pigments. Among these, adding plant extracts before microbial agents has the most significant impact on the synthesis of plastid pigments, particularly β-carotene.

### 3.4 Changes of cellulose and pectin content in cigar leaves

It can be seen from Table 2 that the pectin content in JM1-5 was lower than that in untreated tobacco leaves DX1. Among them, the decrease of pectin content in JM1-3 was significant, and the pectin content in JM3 was the lowest, as low as 0.32 ± 0.26 mg/g of tobacco leaves. The decrease rate of pectin content reached 96%. That is to say, the degradation of pectin is most effective when both media are added simultaneously, and the effectiveness diminishes when added separately. The cellulose content of JM1-5 was significantly lower than that of DX1. Specifically, the cellulose content of JM5 was the lowest at 315.08 ± 42.93 mg/g of tobacco leaf, representing a 46% decrease in cellulose content. The cellulose content of JM3 was lower than that of JM1 and JM2 but higher than that of JM4 and JM5. This suggests that the addition of two media was more beneficial for the degradation of cellulose. Furthermore, the best results were observed when plant extract was added first, followed by microbial inoculants.

**TABLE 2:** pH of the cigar tobacco leaf.

Number	JM1 (d)	JM2 (b)	JM3	JM4	JM5	DX1
pH	6.49 ± 0.01	6.68 ± 0.03	6.24 ± 0.01 f	6.46 ± 0.02 e	6.79 ± 0.01 a	6.53 ± 0.01 c

### 3.5 Aroma components of cigar tobacco leaves

**FIGURE 4:**
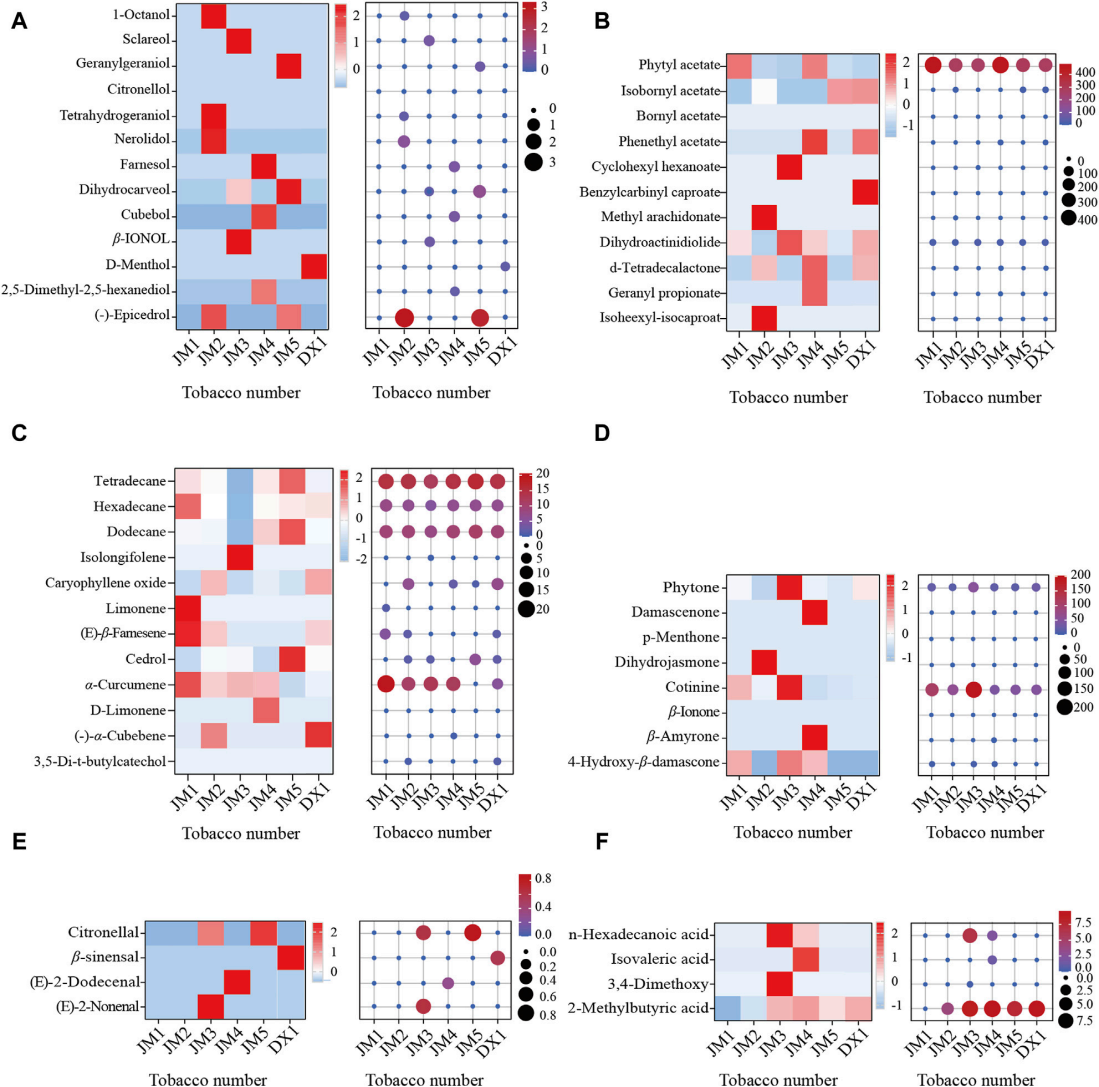
Common aroma components in tobacco leaves. Note: **(A)**: alcoho compounds; **(B)**: ester compounds; **(C)**: other compounds; **(D)**: ketones compounds; **(E)**: aldehyde compounds; **(F)**: acids compounds.

The volatile components of cigar tobacco leaves were determined using GC-MS, and the odorant components were analyzed (Figure 4). It was found that the types and contents of ester substances in DX1 were more abundant. However, after fermentation, alcohol, alkane/olefins, acids, and ketones became more prevalent. The levels of ester substances and ketones were relatively higher in all substances. The results indicated that esters and ketones were dominant in microbial metabolic products. Compared with DX1, the esters decreased in JM1, while the olefins and alkanes increased. Alcohol and ketone levels increased in JM2. The levels of alcohols, ketones, aldehydes, and acids in JM3 were elevated. The levels of alcohols, esters, ketones, and acids in JM4 increased. Alcohols, alkanes, and olefins increased in JM5. The results showed that different mediums had varying effects on the odorant components. The order of adding mediums had different effects on the odorant components, but adding mediums could diversify the odorant components.

### 3.6 Changes of microbial communities in cigar tobacco leaves

The results in Table 3 show that the Sobs index in JM1 was the largest, followed by JM5. This trend is consistent with the Chao1 and Ace indices, indicating that the total number of species in JM1 and JM5 samples was the highest. The Sobs index of JM1 was higher than that of DX1. The Sobs index of JM2 and JM3 is lower than that of DX1; meanwhile, the Sobs index of JM4 and JM5 is higher than that of JM3. This demonstrates that the microbial agent enhances the proliferation of microorganisms in tobacco leaves, while the plant extract suppresses the growth of microorganisms in tobacco leaves. At the same time, the plant extract has an inhibitory effect on the microbial agent, but this effect will be weakened by separate addition. In the Shannon index, JM1, JM2, JM3, and JM5 exhibited higher values compared to DX1, suggesting that the microbial community diversity in these four tobacco leaves was greater than in DX1. JM1 showed higher than DX1; JM1 is higher than JM2 and JM3, indicating that the microbial agent can enhance microorganism diversity more than the plant extract, while the plant extract has an inhibitory effect on the microbial agent. After separate addition, the Shannon value of JM4 decreased significantly and was lower than that of DX1. This could be attributed to the stronger inhibitory effect of post-spraying plant extracts on the microbial agents and the original microorganisms present on tobacco leaves. At the same time, these results are consistent with those reflected by the Simpson index. Table 4.

**TABLE 3:** Cellulose and total pectin content in tobacco.

Tobacco number	Total pectin content/(mg/g)	Cellulose content/(mg/g)
JM1	3.71 ± 0.71 b	482.21 ± 39.42 b
JM2	4.76 ± 1.61 b	371.14 ± 50.41 c
JM3	0.32 ± 0.26 c	364.8 ± 34.81 c
JM4	6.85 ± 1.31 a	336.24 ± 15.65 c
JM5	7.97 ± 0.54 a	315.08 ± 42.93 c
DX1	8.43 ± 0.86 a	583.76 ± 47.64 a

Note: Lowercase letters indicate significant differences between groups, *p* < 0.05.

**TABLE 4:** The α diversity index of microorganism community.

Tobacco number	Sobs	Ace	Chao1	Shannon	Simpson
JM1	5,904	5,904	5,904	4.1871	0.0637
JM2	3,205	3,205	3,205	3.8499	0.0700
JM3	2,760	2,760	2,760	4.1132	0.0463
JM4	4,517	4,517	4,517	1.6122	0.5019
JM5	4,721	4,721	4,721	4.0639	0.0685
DX1	4,618	4,618	4,618	3.7858	0.0890

**FIGURE 5:**
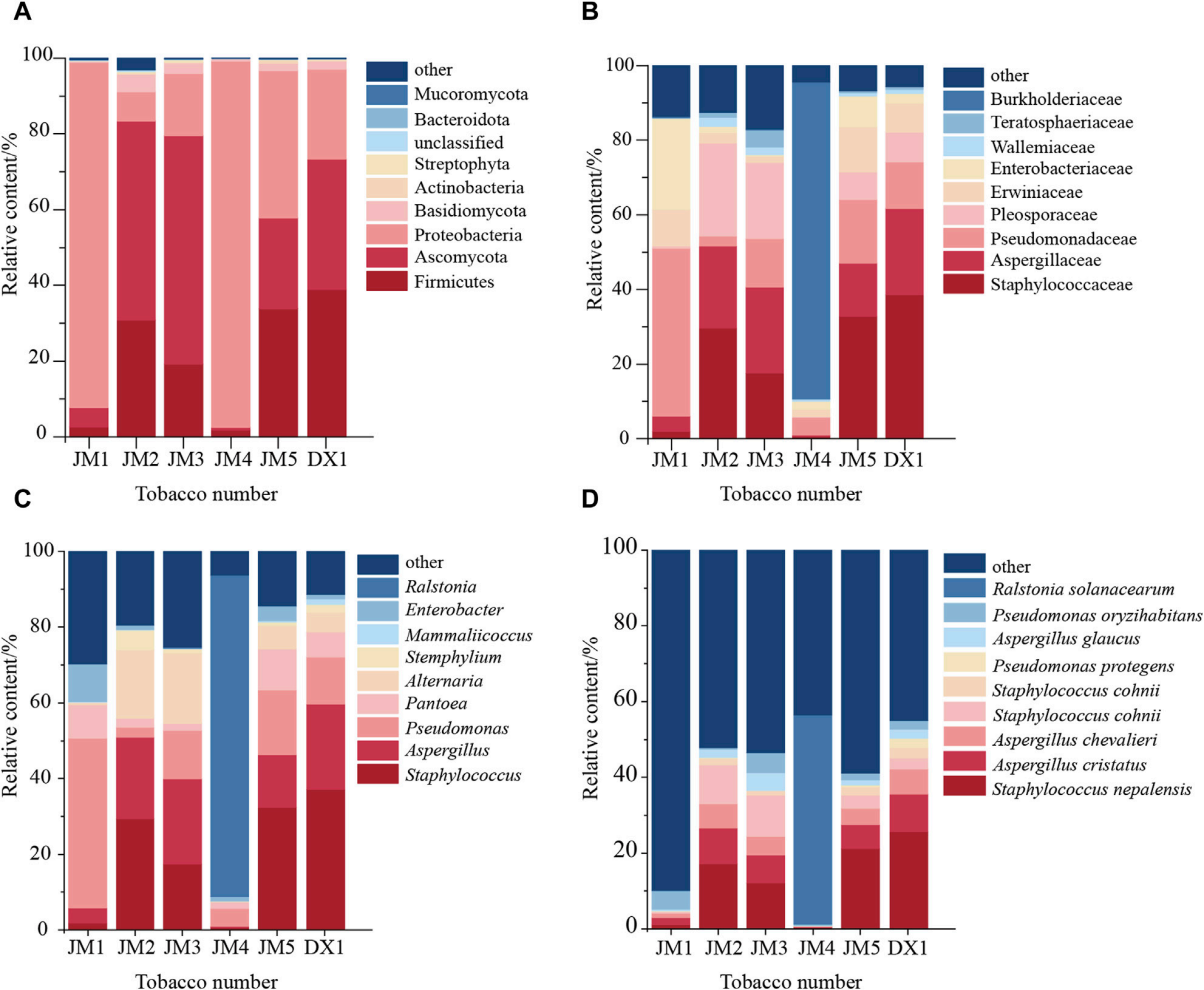
Changes in microbial community in tobacco leaves. Note: **(A)** shows the phylum level; **(B)** shows the family level; **(C)** shows the genus level; **(D)** shows the species level.

Further analysis of the microbial community structure in the samples revealed that at the phylum level (Figure 5A), *Firmicutes* and *Ascomycota* were dominant in DX1 and JM2, JM3, and JM5, while *Proteobacteria* were dominant in JM1 and JM4. Compared with DX1, the proportion of *Proteobacteria* increased in JM5, which had the best evaluation effect, while the proportion of *Ascomycota* decreased. At the genus level (Figure 5C), the species composition of JM4 was the most different from that of other groups. The species composition of JM3 and JM5 was similar to that of DX1, but the proportions were different. The proportion of *Staphylococcus* and *Pantoea* decreased in JM3, while *Alternaria* increased. *Pseudomonas* and *Pantoea* increased in JM5, while *Aspergillus* decreased. At the species level (Figure 5D), compared with DX1, the proportion of *Pseudomonas* protegens decreased in JM5, and *Staphylococcus nepalensis* decreased in JM1-5.

The relative abundance of the top ten microorganisms at the species level was determined for the six samples (Table 5). The top ten microorganisms in DX1 included both fungi and bacteria, with a higher proportion of bacteria. The top ten microorganisms in JM1 are all bacteria, while the species of fungi in JM2 accounted for a higher proportion. Microbial agents promote bacterial growth, while plant extracts promote fungal growth. JM3 is similar to JM2, JM4 is similar to JM1, and JM5 is similar to DX1. This suggests that plant extracts have a direct inhibitory effect on microbial agents, but the inhibitory effect is weakened when added individually.

**TABLE 5:** The top ten relative abundances of microorganisms on the surface of tobacco.

Microorganism		JM1	JM2	JM3	JM4	JM5	DX1
Fungi	*Alternaria alternata*	—	0.1023	0.1079	—	0.0349	0.0290
	*Alternaria tenuissima*	—	0.0279	0.0288	—	—	—
	*Aspergillus chevalieri*	—	0.0645	0.0506	—	0.0431	0.0665
	*Aspergillus cristatus*	—	0.0941	0.0731	—	0.0630	0.0994
	*Aspergillus glaucus*	—	0.0224	0.0470	—	—	—
	*Aspergillus ruber*	—	—	0.0296	—	—	—
	*Hortaea werneckii*	—	—	0.0308	—	—	—
	*Stemphylium lycopersici*	—	0.0509	—	—	—	—
	*Wallemia mellicola*	—	0.0233	—	0.0057	—	—
	Subtotal	0	0.3854	0.3678	0.0057	0.141	0.1949
Bacterial	*Acinetobacter baumannii*	0.0601	—	—	—	—	—
	*Acinetobacter johnsonii*	—	—	—	0.0086	—	—
	*Bacillus altitudinis*	—	—	—	0.0060	—	—
	*Enterobacter cloacae*	0.0245	—	—	—	—	—
	*Enterobacter hormaechei*	0.0411	—	—	0.0036	—	—
	*Kosakonia cowanii*	0.0674	—	—	0.0042	0.0207	0.0011
	*Pantoea agglomerans*	0.0196	—	—	—	0.0151	0.0059
	*Pantoea deleyi*					0.0263	0.0202
	*Pseudomonas oryzihabitans*	0.0494	—	0.0518	—	0.0185	0.0226
	*Pseudomonas parafulva*	0.0196	—	—	0.0105	—	—
	*Pseudomonas psychrotolerans*	0.0347	—	0.0343	—	—	—
	*Pseudomonas putida*	0.0698	—	—	—	—	—
	*Ralstonia pseudosolanacearum*	—	—	—	0.2522	—	—
	*Ralstonia solanacearum*	—	—	—	0.5525	—	—
	*Staphylococcus cohnii*	—	—	—	—	0.0222	0.0277
	*Staphylococcus nepalensis*	—	0.1719	0.1212	0.0033	0.2121	0.2560
	*Staphylococcus warneri*	—	0.0359	—	—	—	—
	*Staphylococcus xylosus*	—	0.0197	—	—	0.0258	0.0202
	unclassified	—	—	—	0.0339	—	—
	Subtotal	0.4339	0.2274	0.2073	0.8749	0.3407	0.3537
	*Bacillus altitudinis*	—	—	—	0.0060	—	—
	*Enterobacter cloacae*	0.0245	—	—	—	—	—
	Total	0.4339	0.6128	0.5751	0.8806	0.4817	0.5486

Note: Indicates undetected.

### 3.7 Changes of carbohydrate active enzyme genes in microorganisms of cigar tobacco leaves

**FIGURE 6:**
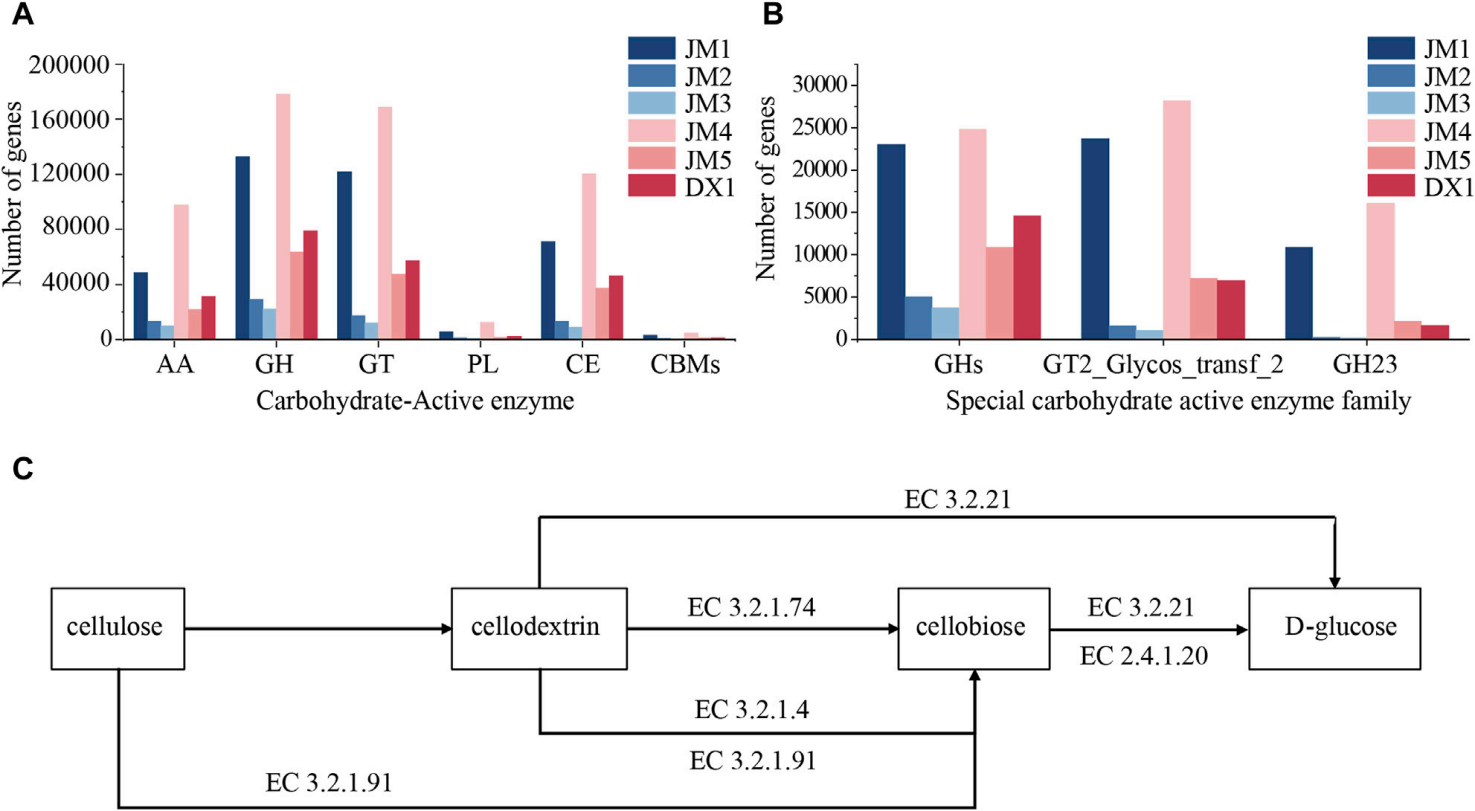
Changes in microbial community in tobacco leaves. **(A)** Content of Carbohydrate active enzyme gene **(B)** Content of Specific carbohydrate active enzyme family gene **(C)** Metabolic pathway of Cellulose.

Carbohydrate active enzyme (Yang, 2021) (CAZymes) is an important enzyme substance for microbial catabolism and utilization of carbohydrates. It includes GH (hydrolysis and/or rearrangement of glycosidic bonds), GT (formation of glycosidic bonds), PL (non-hydrolytic cleavage of glycosidic bonds), CE (esters of hydrolyzed carbohydrates), AA (oxidoreductase synergistic with CAZymes) and CBM (binding to carbohydrates). The carbohydrate active enzyme genes of microorganisms in tobacco leaves were statistically analyzed. It can be seen from Figure 6A that the content of six carbohydrate active enzyme-related genes in JM1 was higher than that in DX1, JM2 was lower than that in DX1, and JM3 was lower than that in JM2, which was consistent with the results of the total amount of microorganisms. The content of carbohydrate active enzyme genes in JM4 was higher than that in JM5 and higher than that in JM1. The abnormal high number of carbohydrate active enzyme genes in JM4 may be due to its higher proportion of bacterial content than other groups and the relative abundance of the top ten microorganisms.

Carbohydrate-active enzymes (CAZymes) are important enzymes for the microbial breakdown and utilization of carbohydrates. It includes GH (hydrolysis and/or rearrangement of glycosidic bonds), GT (formation of glycosidic bonds), PL (non-hydrolytic cleavage of glycosidic bonds), CE (esters of hydrolyzed carbohydrates), AA (oxidoreductase working synergistically with CAZymes), and CBM (carbohydrate-binding). The carbohydrate-active enzyme genes of microorganisms in tobacco leaves were statistically analyzed. It can be seen from Figure 6A that the content of six carbohydrate-active enzyme-related genes in JM1 was higher than that in DX1, JM2 was lower than that in DX1, and JM3 was lower than that in JM2, which was consistent with the results of the total amount of microorganisms. The content of carbohydrate-active enzyme genes in JM4 was higher than that in JM5 and JM1. The abnormally high number of carbohydrate-active enzyme genes in JM4 may be attributed to its higher proportion of bacterial content compared to other groups and the relative abundance of the top ten microorganisms.

## 4 Discussion

This study investigated the impact of various methods of adding plant extracts and microbial agents on the fermentation process. The results of the sensory evaluation indicated that the cigar tobacco leaves developed a distinct aroma following fermentation. The effect of combining two mediums is better than that of a single addition. Among them, the best approach is to add plant extracts first and then microbial agents. The sensation of the cigar is rich, with notes of alcohol, transparent, clean, and sweet.

Studies have shown that β-carotene is one of the carotenoids present in tobacco. Carotenoids serve as crucial precursors of tobacco aroma components, and they are positively correlated with both the quantity and quality of tobacco aroma (Han, 2010; Zhu et al., 2023). The results of the determination of plastid pigment content in tobacco leaves showed that the content of β-carotene was highest in JM3 and JM5, with JM5 slightly higher than JM3. Combined with the scores of aroma characteristics in sensory evaluation results, the relevant findings consistently showed that β-carotene was positively correlated with aroma. The combination of the two media is better than using just one. Among them, the most effective method is adding plant extract first and then adding a bacterial agent, as it can significantly enhance the aroma of tobacco leaves.

Studies have shown that with the change of fermentation time, the microbial community on tobacco leaves will also change correspondingly, and the quality of tobacco leaves will also change (Xiuni et al., 2019; Hu et al., 2023c). In the analysis of microbial community structure, it was found that the microbial composition of JM1 and JM4 was significantly different from that of other groups, in which the proportion of *Firmicutes* was much lower than that of other groups, and the top ten microorganisms with microbial abundance were almost all bacteria. Meanwhile, the content of GH23 in these two groups was significantly higher than that of other groups. The GH23 family includes Chitinase (EC 3.2.1.14), Lysozyme (EC 3.2.1.17), and Peptidoglycan lytic transglycosylase/peptidoglycan lyase (EC 4.2.2.-), which is associated with chitin cleavage. The peptidoglycan content of the cell wall in *Actinobacteria* is high, and chitin is an essential component of the basic structure of fungal cells, constituting up to 45% of the dry weight of fungi (Liu et al., 2020). The biofertilizer stimulates the growth of bacteria with GH23-related genes, consequently inhibiting fungal growth. However, plant extracts counteract the effects of the biofertilizer.

In this study, *Firmicutes* and *Ascomycetes* were the dominant bacteria in JM5 tobacco leaves. The proportion of *Pseudomonas* and *Panthenium* in JM5 increased, which may be related to the improvement of tobacco leaf quality. Compared with DX1, the proportion of *Pseudomonas protegens* (known for rapid growth rate and metabolism) in JM5 decreased. Studies (Garrido et al., 2023) have shown that additional inoculation of *P. protegens* would affect the composition of the original rhizosphere microorganisms in wheat. That is, the addition of bacterial agents and plant extracts may inhibit the growth of *Pseudomonas* protegens, and then promote the co-growth of other microorganisms. The proportion of *Aspergillus* in JM5 is reduced, especially *Aspergillus glaucus* [one of the main fungi causing agricultural product loss (Yuan et al., 2020)]. The reduction of Aspergillus glaucus proportion may reduce the risk of mildew to a certain extent.

Cellulose is a crucial component of tobacco material, serving as the framework of tobacco cell structure and impacting tobacco smoking quality with noticeable irritation and impurities (Yan et al., 2016; Zhu et al., 2016). Research has shown that *Pseudomonas putida* can efficiently break down and utilize lignocellulose (Elmore et al., 2020), leading to the production of valuable chemicals from lignin and D-glucose (Kohlstedt et al., 2018). The presence of *P. putida* is higher in JM1 and JM5 compared to DX1, with lower cellulose content in both, suggesting a potential link between *P. putida* and reduced cellulose content in tobacco leaves. Mold-produced mycelium can infiltrate cellulose, hemicellulose, and lignin, while mold-secreted cellulase can degrade cellulose effectively from within, displaying strong degradation capabilities. Various microbial genera, including *Aspergillus* (Li et al., 2019), have been identified to possess cellulose degradation functions. The proportion of *Aspergillus* in JM2 and JM3 is higher than that in DX1, so the related *Aspergillus* may be associated with the decrease of cellulose in JM2 and JM3 tobacco leaves. Cellulose is also a carbohydrate in nature, and its decomposition relies on the microorganisms’ own carbohydrate-active enzymes. The active carbohydrate enzyme family related to cellulose synthesis is GT2_Glycos_transf_2[CDD Conserved Protein Domain Family: Glycos_transf_2 (nih.gov)], and the encoding genes of glycoside hydrolase related to cellulose degradation are distributed in seven glycoside hydrolase families (GH1, GH3, GH5, GH6, GH7, GH12, and GH45) (Xing et al., 2022). The number of these genes in JM4 is higher than that in DX1, which may be linked to the decrease of cellulose content in JM4. In conclusion, adding bactericide and plant extract to fermented tobacco leaves can reduce the fiber content in tobacco leaves. The bactericide may achieve its purpose by increasing *P. putida*, while the plant extract may increase the proportion of *Aspergillus*. Simultaneously, plant extracts can significantly inhibit the effect of bacterial agents when added together, and the inhibitory effect is weakened when added separately. Adding plant extracts before adding bacterial agents has the best effect on the degradation of cellulose in tobacco leaves.

## 5 Conclusion

Different medium and the order of medium addition have significant effects on the fermentation process of tobacco leaves. The best medium is a combination of plant extract and bacterial agent. The best method of application is to first spray plant extract and then spray the bacterial agent alone. The fermentation process enhances the aroma of the cigar, making it cleaner and sweeter. It also helps to reduce the cellulose content in tobacco leaves by up to 46%. The addition of β-carotene can significantly enhance the aroma of cigar by increasing its content. This results in an increase in aroma components such as alcohols, alkanes, and olefins in tobacco leaves. Moreover, the microbial community structure of tobacco leaves undergoes changes, with an increase in the proportion of *Pseudomonas* and *Pantoea*, and a decrease in the proportion of *Aspergillus*.

## Data Availability

The data presented in the study are deposited in the SRA database, accession number PRJNA1141179. Available at: https://www.ncbi.nlm.nih.gov/sra/PRJNA1141179.
